# Reconstruction plate versus minimal invasive retrograde titanium elastic nail fixation for displaced midclavicular fractures

**DOI:** 10.1007/s10195-011-0158-7

**Published:** 2011-09-27

**Authors:** Jamal E. H. Assobhi

**Affiliations:** Faculty of Medicine for Girls, Al-Zahraa University Hospital, Al-Azhar University, 5 Al-Emam Al-Sha’rawy str., Assefarat District, Nasr City, Cairo, Egypt

**Keywords:** Midclavicular, Fractures, Titanium, Elastic, Nail, Plate

## Abstract

**Background:**

Nonoperative management of midshaft clavicle fractures (MSCFs) is standard; however, it is now generally accepted that displaced midshaft clavicle fractures benefit from internal fixation. Plating and intramedullary fixation have become the accepted methods of fixation. The purpose of this study was to see if one method of fixation of clavicle fractures has a lower complication rate and higher union rate than the other.

**Materials and methods:**

Between December 2003 and September 2008, 38 patients were treated randomly by either plating (plate group) or retrograde nailing (RTEN group). Primary outcome measures included functional Constant scores, radiological union rate and union time. Clinical and radiological assessments were performed at the 6th week and the 3rd, 6th and 12th month postoperatively. Secondary outcome measures included the perioperative data (mean surgery time, blood loss, wound size, and hospital stay), and the complication rates.

**Results:**

Similar results were found between the two groups regarding functional and radiological outcome after the 12th week (*P* > 0.05). However, earlier union and functional recovery were obtained at the 6th week for the RTEN group (*P* < 0.05). The rate of complications was significantly higher (15.8%) in the plate group compared with the RTEN group (0%; *P* > 0.05). In the plate group, significantly higher values were obtained for the perioperative data (*P* < 0.001).

**Conclusion:**

Both techniques are equally effective at treating displaced midclavicular fractures, and give better function and fewer complications than nonoperative treatment. The RTEN technique has more advantages and lower complications than plating, making its use more favorable. It is recommended for athletes and young active individuals, and can be used as an alternative to conservative treatment or plate fixation.

## Introduction

Fractures of the clavicle have been reported to represent 2.6% of all fractures [23]. The midshaft is the most frequently affected site, encompassing 69–82% of all clavicle fractures, and most fractures that occur in the midshaft are displaced [25, 27]. Midshaft clavicle fractures (MSCFs) in adults have traditionally been treated nonoperatively [12, 18, 27]. However, displaced or comminuted fractures carry a risk of symptomatic malunion, nonunion, and poor functional outcome with cosmetic deformity [3, 9, 31]. Recent studies have reported a rate of nonunion of 15% [9] following nonsurgical intervention. Early surgical intervention of MSCFs has resulted in improved outcomes and a decreased rate of nonunion and symptomatic malunion compared with nonoperative treatment [3, 6, 9]. However, the optimal treatment for isolated acute displaced MSCF remains controversial [31, 32].

Operative treatment of displaced MSCFs can be achieved successfully using plates [1, 22] or intramedullary (IM) implants like Rush pins [7], Kirschner wires [19], or nails [20]. However, the use of the later rigid IM implants resulted in serious complications, such as intrathoracic migration and damage to the underlying structures [16, 21]. Recently, Jubel et al. [13] introduced a new IM nailing technique in which a single titanium elastic nail (TEN) is inserted in an antegrade manner from the sternal end of the clavicle to fix those fractures. He reported fewer complications and a higher rate of fracture healing than those previously reported with the use of rigid IM implants. Rehm et al. [24] treated 136 displaced MSCFs successfully using the same antegrade fixation technique with a single TEN. However, a risk of iatrogenic perforation of the lateral cortex in 5 patients was reported.

This prospective randomized study was designed to assess the effectiveness of minimally invasive retrograde titanium elastic nailing (RTEN) for the treatment of displaced MSCFs, and to compare its outcome with that of a standard anteroinferior plating technique.

## Materials and methods

The study was approved by the local ethical committee and performed in accordance with the ethical standards of the 1964 Declaration of Helsinki as revised in 2000. All patients gave their informed consent.

In a prospectively randomized study, between December 2003 and September 2008, a total of 38 patients with displaced MSCFs were randomized into two equal groups to be treated surgically with either a 3.5 mm reconstruction plate located anteroinferiorly to the clavicle (plate group) or with a single TEN fixation in a retrograde mode (RTEN group). The characteristics of the patients of both groups are shown in Table 1. Lehr’s formula was used for the sample size calculation [15]. The required sample size, after setting the power to 80% to detect a Constant score difference of 10 as being statistically significant at the 5% level, was 38. Each group required at least 19 participants. Patients were randomized into two groups by the concealed envelope technique.

**Table 1:** Characteristics of the patients of both groups

	Plate group	RTEN group
Male:female	17:2	16:3
Dominant:nondominant	16:3	15:4
Right:left	14:5	12:7
Average age (years)	32.6 ± 5.9 (range 26–49)	30.3 ± 4.8 (range 24–45)
Cause of injury		
Vehicle accidents	7	9
Sporting activities	4	5
Fall from height	8	5

*Inclusion and exclusion criteria.* Patients were included in the study if their ages were between 16 and 60 years of age and they had suffered displaced midshaft clavicular fractures within the last 4 weeks with no cortical bone contact or shortening of over 15 mm, or if the fracture fragments were tenting or compromising the skin with an axial malalignment of over 30°. Patients were excluded if they had fractures with marked comminution or that were older than 4 weeks, ipsilateral injuries that could influence the recovery and the scoring systems, pathological fractures, open fractures, a congenital anomaly or bone disease, or if there was cellulites around the incision site for open reduction.

### Surgical techniques

**Fig. 1:**
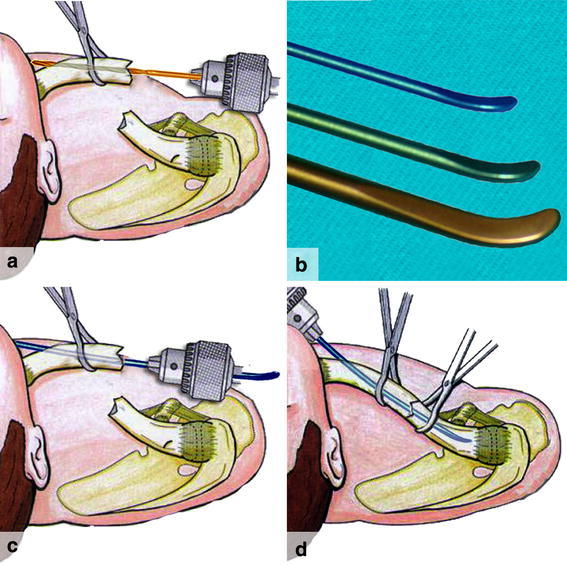
The principle of the open retrograde TEN fixation technique. **a** Top view of a right shoulder shows that the medial fragment is held away from the fracture by bone forceps, and drilling is aimed obliquely at its anterior cortex. **b** The common diameters of the titanium elastic nails that are suitable for clavicle fixation are, *from top to bottom*, 2, 2.5, and 3 mm. **c** Insertion of the nail through the reamed canal until it is out of the anterior cortex. The nail is withdrawn from its back end until the curved tip is lying flush to the fracture surface. **d** The fracture is then reduced and the nail is pushed inside the medullary canal of the lateral fragment

The principle of the retrograde nailing technique is similar to that described for Kuntscher’s open IM nailing of the clavicle (Fig. 1) [20]. After general anesthesia, the patients of both groups received 1 g of intravenous cefazolin as a prophylactic antibiotic. They were all positioned in the beach-chair position with a folded towel under the affected shoulder. The affected upper extremity was draped free to allow manipulation in a sterile manner. The image and its monitor were placed in front of the surgeon on the opposite side of the operating table so that perpendicular shots and those with 20–45° of cephalic tilt could be taken to view the I- and S-shaped forms of the clavicle, respectively [30].

#### The RTEN surgical technique

**Fig. 2:**
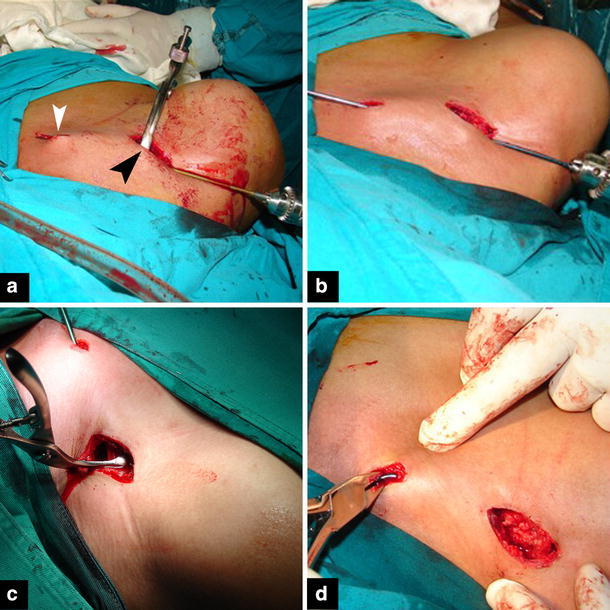
Operative photos of the RTEN technique. **a** A small incision is made over the fracture site (*black arrowhead*). The medial fragment is grasped with bone forceps and lifted up in the wound. The medullary canal is reamed with a drill bit with a diameter similar to the nail (usually a 2.5 mm bit is required). When the drill bit has penetrated the anterior cortex and is felt under the skin, a tiny incision is made over it. **b** A nail is inserted through the medullary canal and allowed to come out through the small medial incision. **c** The fracture is reduced and the nail is pushed inside the lateral fragment under fluoroscopic control. **d** Bending of the tip of the nail before wound closure

A small incision 3–4 cm long is made over the fracture site to enable direct manipulation of the fragments (Fig. 2a). A minimal amount of soft tissue and periosteum is then released. The medial fragment is grasped with small bone holding forceps and lifted out of the wound. A drill bit with a diameter that is similar to that of the proposed nail diameter is inserted inside the medullary canal of the medial fragment, pointing slightly anteriorly to penetrate the anterior cortex. When the tip of the drill bit is felt beneath the skin, another tiny skin incision is made over it (Fig. 2a). The nail, which is fixed to a universal chuck with a T-handle, is passed retrograde across the fracture into the predrilled medullary canal under fluoroscopic control and allowed to exit from the medial incision (Fig. 2b). The protruded end is grasped again by a universal chuck and the nail is pulled medially until clear of the fracture, which is then reduced (Fig. 2c). The nail is next driven across the fracture site into the medullary canal of the lateral fragment until resistance is felt by the surgeon. To ensure the correct placement and depth of the nail into the lateral fragment, fluoroscopic control is used. The protruding end of the nail is cut off and bent as close to the bone as possible (Fig. 2d). Using the impactor, the bent end of the nail is impacted and the skin is closed over the bent end of the nail.

#### The anteroinferior plating

**Fig. 3:**
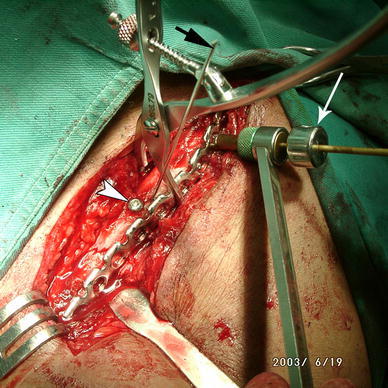
Plate fixation technique. The plate is contoured in an S-shape manner to fit the clavicle curvature. A k-wire (*black arrowhead*) can be used for temporary fracture fixation until a lag screw (*white arrowhead*) is inserted away from the plate in the oblique fracture. While drilling the screws through the plate holes, a drill stopper (*white arrow*) is used to avoid injury to neurovascular structures

An incision is made parallel to the inferior border of the clavicle, and dissection is carried out down to the fracture site, followed by careful subperiosteal dissection [4, 22]. The fracture is reduced and held temporarily with bone clamps. An adequate plate length of the 3.5-mm reconstruction plate is contoured to adapt to the anterior S-shape of the clavicle, as recommended by Kloen et al. [14] and Collinge et al. [4]. In most cases, a six- to eight-hole reconstruction plate is required when contoured into an S-shape. A cortical lag screw is usually fixed across the fracture site in oblique fractures. When drilling the screw holes, a drill stopper is required in order to avoid injury to neurovascular structures (Fig. 3).

For both groups, patients received arm sling protection for 1–2 weeks postoperatively, and then light daily activities such as writing or eating were allowed. Patients were encouraged to resume their normal daily activities after the 4th week when the pain was tolerated. Strenuous activities were discouraged before the 6th week from the trauma.

### Assessment

Following hospital discharge, patients underwent clinical and radiological evaluations at regular follow-up visits (FUV) at the 6th week and at the 3rd, 6th and 12th months postoperatively to assess fracture healing and function of the arm, and to record any complication.

#### The primary outcome measure

*Shoulder function* was evaluated according to the 100-point scoring system of Constant and Murley [5]. This system combines assessments of subjective symptoms and objective findings. The overall grading is excellent if the total score ranges from 90 to 100, good for 80–89, fair for 70–79, and poor if the scores are 69 or less [11]. Radiographic healing was defined as evidence of bridging callus or obliteration of the fracture lines. Clinical union was considered an absence of tenderness at the fracture site. Time to heal was then recorded when all of these criteria were fulfilled.

#### Secondary outcome measures

##### Perioperative data

Operative time, amount of blood loss, and size of the surgical wound were recorded for every patient. The difference in the weights of the sponges pre- and postoperatively and the added volumes of suction were recorded to determine the amount of blood loss.

##### Complication rates

Complications such as nonunion, implant failure, wound infection, thoracic outlet syndrome, and refracture after implant removal were recorded.

##### Cosmetic outcome

At the end of the follow-up period (12th month), cosmetic outcome was assessed. The cosmetic appearance was determined with special regard to asymmetry of the shoulder, visible deformity, hypertrophic scars, or prominence of the implant under the skin. Asymmetry of the shoulder was determined by measuring the distance from the center of the jugular fossa to the lateral tip of the acromion. In comparison with the intact contralateral side, a difference of greater than 0.5 cm was considered to be significant asymmetry.

#### Statistical analysis

To evaluate the significance of the functional assessment of Constant scores across time, a generalized linear model (GLM) repeated measures analysis of variance (ANOVA) was used. The perioperative data and the average bone union time were compared with the unpaired nonparametric Mann–Whitney test. Fisher’s exact test was used to compare the incidence rates of the two groups. All tests were done using Statistica version 6.0 and Graphpad Instat version 3.06 statistical software. *P* values < 0.05 were considered significant.

## Results

Surgeries were performed at a mean of 9.5 days ± 5.9 days (range 1–22 days; median, 9 days) after injury for the plate group and a mean of 10.2 days ± 6.2 days (range 1–23 days; median, 9 days) after injury for the RTEN group. In 16 patients, a nail diameter of 2.5 mm was used, while in 3 patients the 3 mm nail was used. The mean follow-up period was 18.6 ± 3.8 months (range 14–26 months) for the plate group and 14.5 ± 1.5 months (range 12–18 months) for the RTEN group.

### The primary outcome results

Assessment at the 6th week showed significant higher Constant scores of 69.1 ± 9.2 (with a 95% confidence interval of 64.7–73.5) in the RTEN group than in the plate group [62.1 ± 11.4 (*P* < 0.05) with a 95% confidence interval of 64.7–73.5]. Otherwise, similar results for the two groups were found at the subsequent assessment periods (*P* > 0.05), as shown in Table 2.

**Table 2:** Average constant scores between groups across time

Assessment period	Group A mean ± SD^‡^	95% confidence interval (CI)		Group B mean ± SD	95% confidence interval (CI)		*P* value^Ψ^
		LCI	UCI		LCI	UCI	
6 weeks	62.1 ± 11.4	56.6	67.6	69.1 ± 9.2	64.7	73.5	0.023*
3 months	77.3 ± 10.3	72.3	82.2	83.0 ± 8.7	78.8	87.2	0.071
6 months	84.7 ± 12.2	78.8	90.6	90.3 ± 7.3	86.8	93.8	0.078
12 months	89.9 ± 11.3	84.4	95.3	95.5 ± 5.3	92.9	98.1	0.076

*SD* standard deviation, ^Ψ ^stat test = repeated measures GLM ANOVA

At final evaluation, the overall grading of the results in the RTEN group was 17 excellent and 2 good, while in the plate group there were 14 excellent, 2 good, 1 fair, and 2 unsatisfactory results.

**Fig. 4:**
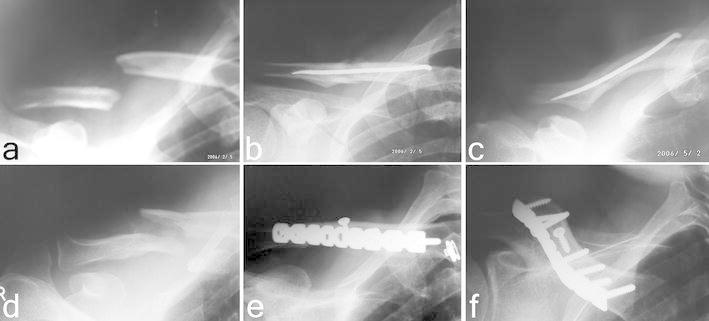
Radiological assessment of surgically treated groups. **a** A preoperative X-ray of a 33 year old heavy manual laborer in the RTEN group. **b** Immediate postoperative anteroposterior radiograph showing good alignment. **c** Follow-up radiograph at the 3rd month at 45° cephalic tilt showing complete bone consolidation. **d** A preoperative radiograph of a 40 year old heavy manual laborer with a displaced midshaft clavicular fracture with some comminution (patient was in the plate group). **e** Immediate postoperative anteroposterior radiograph showing good alignment. **f** Follow-up radiograph with complete bone union at 6 months

The average bone union time was significantly shorter in the RTEN group (5.2 months ± 1.7; range 3–9 months) than in the plate group (7.3 months ± 3.1; range 3–12 months) (*P* = 0.034) (Fig. 4).

### Secondary outcome results

#### Immediate results

Significant higher values were obtained in the plate group than in the nail group regarding mean operative time, blood loss, size of surgical wound, and length of hospital stay (*P* < 0.001) (Table 3).

**Table 3:** Perioperative data comparisons between groups

Characteristics	Group A (*N* = 19)	Group B (*N* = 19)	*P* value^ψ^
	Mean ± SD (range)	Mean ± SD (range)	
Duration of surgery (min)	68.1 ± 10.9 (54–92)	44.1 ± 9.1 (28–64)	0.0001
Mean wound size (cm)	8.5 ± 1.6 (6–11)	4.3 ± 0.8 (3–6)	0.0001
Average blood loss (ml)	144.3 ± 31.5 (89–195)	65.4 ± 28 (27–100)	0.0001
Mean hospital stay (days)	2.3 ± 0.8 (1–4)	1.4 ± 0.5 (1–2)	0.0007

*SD* standard deviation, ^ψ^ unpaired nonparametric Mann–Whitney test

#### Complications

In general, no statistically significant differences were found between the incidences of complications in the groups (Table 4). Three complications (15.8%) were encountered in the plate group: nonunion in 1 (5.3%), infection in 1 (5.3%), and refracture in 1 (5.3%). No complications were encountered in the RTEN group.

**Table 4:** Major and cosmetic complications

Complication	Plate (*N* = 19)		RTEN (*N* = 19)		*P* value^Ψ^
	No	Rate (%)	No	Rate (%)	
Major					
Nonunion	1	5.3	0	0.0	1.00
Implant failure	0	0.0	0	0.0	1.00
Wound infection	1	5.3	0	0.0	1.00
Thoracic outlet syndrome	0	0.0	0	0.0	1.00
Refracture after implant removal	1	5.3	0	0.0	1.00
Total major complications	3	15.8	0	0.0	0.23
Cosmetic					
Asymmetry	0	0.0	0	0.0	1.00
Hypertrophic callus	0	0.0	1	5.3	1.00
Hypertrophic scar	4	21.1	0	0.0	0.11
Prominent implant under skin	3	15.8	3	15.8	0.23
Total cosmetic	7	36.8	4	21.1	0.48

^Ψ^Fisher’s exact test was used

#### Cosmetic results

There were 4 patients (21.1%) with hypertrophic scars and 3 patients (15.8%) had prominent implants under the skin in the plate group. In the RTEN group, one patient (5.3%) had asymptomatic hypertrophic callus and another 3 patients (15.8%) had a prominence at the cut bent end of the nail.

## Discussion

Although most middle-third clavicular fractures can be treated nonoperatively, several recent studies have demonstrated a poorer outcome in association with displaced, comminuted midshaft fractures that were treated nonoperatively [9, 26, 31]. Therefore, a selective surgical treatment for a midclavicular fracture is recommended [1, 12, 13].

In a recent randomized, prospective study in which nonoperative treatment of displaced midshaft clavicular fractures was compared with plate fixation, patients in the operative fixation group had significantly better functional outcomes, a lower rate of nonunion, and a lower incidence of symptomatic malunion [3]. Most complications in the operative fixation group were hardware related.

Currently, plate fixation [14, 28] and IM titanium elastic nailing [13, 24] are the available methods for the treatment of severely displaced MSCFs. The options for plate location are anteroinferior and superior [4, 10, 12, 14]. The theoretical advantages of anteroinferior plating include less hardware prominence and the ability of the surgeon to direct instrumentation away from infraclavicular neurovascular structures [4, 14].

In this study, anteroinferior plating was compared with retrograde IM nailing for displaced MSCFs in adults.

The reported 31% risk of unsatisfactory results following nonoperative treatment in the Hill et al. study [9] was reduced in this study to 2/38 (5.3%) as a result of surgical intervention. The 15% [9] risk of nonunion in that study was also reduced to 2.6% in ours as a result of surgical intervention in both groups in this study.

Comparing the results of plating in this study with those reported in the literature, infection was found in one (5.5%), while the reported rates in the literature range from 0 to 18% [1–3]. However, infection was eventually controlled after plate removal in the eleventh month when X-rays showed evidence of bone union.

The results of plating compared favorably with those reported in the literature [4]. Satisfactory results were obtained in 17 patients (89.5%) and unsatisfactory results in 2 (10.5%); nonunion was observed in one, and persistence of infection with implant loosening in another one. In the former, bone grafting was carried out in the presence of the implant, and in the latter, removal of the implant and surgical wound debridement was required to control infection. In both cases, bone union was achieved 3 months later.

The reported incidence of refracture following plate removal ranged from 0 to 8% [2, 22]. In this study, after the final follow-up, refracture occurred in one female obese patient who fell following plate removal. Fortunately, the fracture was undisplaced and no surgical intervention was required until the fracture had healed.

The use of the anteroinferior site for the 3.5 mm reconstruction plating allowed adequate fixation of the lateral fragment. This was facilitated by its easier contouring to fit the S shape of the clavicle than for other plates, thereby providing maximum fracture stability. The technique provides stable fixation, avoids risk to the vital structures below the clavicle, and has a low rate of implant prominence and low incidences of nonunion and implant failure.

The large wound size and extensive soft tissue stripping required for plate fixation and removal are disadvantages of plating techniques [1, 2]. In this study, the removal of plates necessitated new admissions, general anesthesia, and another large-sized incision, while nail removal was performed as an OPD under local anesthesia with minimal sedation and a tiny incision over the tip of the nail.

Intramedullary fixation is an attractive alternative that is much less invasive and avoids most of the problems encountered with plating. The clavicle, which is similar to other long bones, is usually best treated with IM methods [13, 24, 29].

Because of anatomic features of the clavicle [17], devices for IM fixation need to be flexible. Aside from the need for flexibility, the implant diameter needs to be small enough to enable its passage through the medullary space, which is narrow, especially in the middle third of the clavicle. Both its elasticity and small diameter mean that IM positioning is ideal and provides good stability [8]. In this study, the nail diameters required for fixation were the 2.5 mm and the 3 mm, and these resulted in perfect adaptation to the complex anatomical shape of the clavicle with good stability. It has been clearly shown that RTEN fixation provides stable fixation and allows an earlier return to normal activities, as well as complete bone healing in all cases without major complications, in contrast with plate fixation. The present RTEN technique allowed the tip of the bent cut end of the nail to migrate only subcutaneously during the course of bone healing. Prominence of the tip of the nail occurred in 3 cases after the bone had healed, and the nails were removed under local infiltration anesthesia as an OPD procedure. This minimally invasive technique provides less soft tissue stripping than the plating technique and results in good biological fracture healing.

Both the anteroinferior plating and IM RTEN fixation techniques are equally effective for the treatment of acute displaced mid-shaft clavicular fractures. However, the present RTEN technique is simpler and quicker, is minimally invasive with less soft tissue injury, requires a shorter operating time, involves less blood loss, results in a shorter hospital stay, leads to faster bone union with 100% union rate, allows an earlier return to normal activities, and produces an excellent cosmetic outcome. In addition, if the implant needs to be removed, this is done under local anesthesia, with a tiny skin incision made over the tip of the nail.

The RTEN technique is recommended for the fixation of displaced mid-shaft clavicular fractures, especially for young active individuals or heavy manual laborers. It can be used as an alternative to conservative treatment or plate fixation.
